# 免疫检查点抑制剂风湿性毒副反应诊治建议

**DOI:** 10.3779/j.issn.1009-3419.2019.10.12

**Published:** 2019-10-20

**Authors:** 佳鑫 周, 迁 王, 炼 段, 晓燕 斯, 丽 张, 小伟 刘, 玥 李, 汉萍 王, 潇潇 郭, 文 张, 力 张

**Affiliations:** 1 100730 北京，中国医学科学院，北京协和医院风湿免疫科 Department of Rheumatology, Peking Union Medical College Hospital, Chinese Academy of Medical Sciences, Beijing 100730, China; 2 100730 北京，中国医学科学院，北京协和医院内分泌科 Department of Endocrinology, Peking Union Medical College Hospital, Chinese Academy of Medical Sciences, Beijing 100730, China; 3 100730 北京，中国医学科学院，北京协和医院呼吸内科 Department of Respiratory medicine, Peking Union Medical College Hospital, Chinese Academy of Medical Sciences, Beijing 100730, China; 4 100730 北京，中国医学科学院，北京协和医院检验科 Department of Clinical, Laboratory, Peking Union Medical College Hospital, Chinese Academy of Medical Sciences, Beijing 100730, China; 5 100730 北京，中国医学科学院，北京协和医院眼科 Department of Ophthalmology, Peking Union Medical College Hospital, Chinese Academy of Medical Sciences, Beijing 100730, China; 6 100730 北京，中国医学科学院，北京协和医院消化内科 Department of Gastroenterology, Peking Union Medical College Hospital, Chinese Academy of Medical Sciences, Beijing 100730, China; 7 100730 北京，中国医学科学院，北京协和医院心内科 Department of Cardiology, Peking Union Medical College Hospital, Chinese Academy of Medical Sciences, Beijing 100730, China

**Keywords:** 免疫检查点抑制剂, 免疫相关副反应, 风湿性疾病, 治疗, Immune checkpoint inhibitors, Immune-related adverse events, Rheumatic disease, Treatment

## Abstract

近年来，免疫检查点抑制剂（immune checkpoint inhibitors, ICIs）已成为肿瘤治疗的突破性进展之一。但随着ICIs的广泛应用，这类药物所导致的免疫相关副反应（immune-related adverse events, irAEs）也逐渐引起人们的重视。研究发现，irAEs几乎可影响到人体的各个器官。本文主要针对ICIs在风湿方面毒副反应的临床特点展开综述，并且总结了具有基础风湿病的患者在使用ICIs后的免疫相关副反应，最后对这类副反应的治疗进行了探讨。

## Abstract (en)

Immune checkpoint inhibitors (ICIs) have made remarkable breakthroughs in cancer treatment. However, the widely use of ICIs is associated with a spectrum of immune-related adverse events (irAEs). These adverse events can affect any organ system. In this article, we have made a systemic review about the clinical characteristics of rheumatic irAEs, and also summarized irAEs in patients with pre-exsiting rheumatic disease. We also focus on the management of rheumatic irAEs.

免疫检查点抑制剂（immune checkpoint inhibitors, ICIs）为近年来肿瘤治疗的亮点之一，通过阻断对T细胞的负性共刺激作用，可诱导T细胞的活化，从而增强其抗肿瘤作用，并改善了一些肿瘤患者的生存状况^[1]^。目前研究已发现有多种免疫检查点抑制剂作用通路，但已在临床中应用的ICIs其主要作用的通路为程序性细胞凋亡蛋白-1（programmed cell death receptor-1, PD-1）通路，主要包括PD-1抑制剂和PD-1配体1（programmed cell death ligand-1, PD-L1）抑制剂，以及抗细胞毒T淋巴细胞相关抗原-4（Cytotoxic T lymphocyte associated antigen 4, CTLA-4）通路（CTLA-4抑制剂）^[2, 3]^。然而，从另一个角度来看，这类治疗可以影响人体正常组织的免疫耐受，从而导致免疫相关的副反应（immune-related adverse events, irAEs）^[4]^。目前的研究发现，irAEs几乎可影响到人体的各个器官，而关于irAEs的研究也吸引了除了肿瘤科医生之外的其他各专科医生。

对于大部分风湿性疾病而言，其主要的发病机制为免疫系统的异常激活导致自身抗体的产生或机体炎症反应的增强。以类风湿关节炎（rheumatoid arthritis, RA）为例，有研究^[5]^发现尽管在RA患者滑膜组织浸润的T细胞中PD-1的表达增加，但是整个PD-1通路在RA患者中是下调的，提示PD-1通路在RA的发病过程中占有一定的地位。而CTLA通路亦在RA的发病过程中起着重要的作用，目前已在国外上市的治疗RA的药物阿巴西普（Abatacept）即为CTLA-4和人免疫球蛋白1（IgG1）Fc段的融合蛋白^[6]^。也曾有研究^[7]^发现在小鼠动物模型中PD-1通路可有助于预防小鼠出现狼疮样的症状。因此，研究ICIs和风湿性疾病的关系也引起了科研人员的注意。从目前各方面的研究结果来看，风湿方面的irAEs主要可分为两大类，第一类为患者使用ICIs之后出现新发的骨关节肌肉症状或者结缔组织病，第二类则为已有风湿性疾病或结缔组织病的患者在使用ICIs之后出现原有疾病的再发或加重。以一项法国单中心的前瞻性研究^[8]^为例，该研究一共纳入了524例接受ICIs治疗的患者，其中有35例（6.6%）患者出现了风湿方面的irAEs，从接受ICIs治疗到出现上述症状的平均时间为70 d。从临床表现来看，有15例（2.9%）患者出现了非炎症性关节肌肉症状，11例（1.9%）患者可诊断为风湿性多肌痛（polymyalgia rheumatica, PMR），7例（1.3%）符合RA的诊断。研究发现风湿方面irAEs最常见的症状仍是关节痛和肌痛，由于这些症状有时相对较轻，在临床工作或研究中有可能会被忽视，因此需要提醒临床医生们重视风湿方面的irAEs。在下文中，我们将详细介绍ICIs在风湿方面的副作用及相应的诊治处理。

## 1 PMR/巨细胞动脉炎（giant cell arteritis, GCA）

PMR主要见于年龄 > 50岁的人群中，其典型的临床表现为肢带肌疼痛和僵硬，辅助检查可有炎症指标的明显升高，类风湿因子（rheumatoid factor, RF）和抗环瓜氨酸多肽抗体（anti-citrulline polypeptide antibody, ACPA）通常为阴性，且患者通常对小剂量激素的反应良好。GCA是一类以侵犯大动脉为主并以血管内层弹性蛋白为中心的坏死性动脉炎，伴肉芽肿形成，在我国相对罕见^[9]^。由于GCA患者常合并有PMR，因此常将这两种疾病一起讨论。

对于使用ICIs后所导致的PMR，目前文献报道从使用ICIs到发病的时间为10 d-1年不等，其临床及影像学表现与传统的风湿性多肌痛相同，但不同的是这部分患者的炎症指标可能是正常的，且有部分患者对小剂量激素的反应不佳^[10]^。少数患者在使用ICIs之后还有可能出现GCA，临床症状包括头痛、颞动脉触痛、下颌间歇性跛行、视力下降等，其病理表现与传统的GCA所示一致^[11, 12]^。

## 2 炎性关节炎（inflammatory arthritis, IA）

IA也是肿瘤患者应用ICIs之后常出现的风湿方面irAEs之一，在多项研究中均有报道，从应用ICIs到出现IA的时间为2个月-24个月不等，其严重程度也不一致，轻者应用非甾类抗炎药（non-steroidal anti-inflammatory drugs, NSAIDs）或小剂量激素后即能得到缓解，重者则有可能需要应用肿瘤坏死因子（tumor necrosis factor, TNF）抑制物或者白介素-6（interleukin-6, IL-6）受体抗体^[13-15]^。ICIs所导致的IA主要可以分为两大类，一类主要类似于RA的表现，受累关节以小关节为主（近端指间关节、掌指关节和腕关节等），可出现骨侵蚀。但与传统RA不太一致的是，这类患者在流行病学上并非多见于女性，且患者血清RF和ACPA通常为阴性，并且患者的骨侵蚀通常发生要更早。临床中也有少数患者表现为ACPA阳性的RA，不过这些患者中有部分在使用ICIs之前即已有ACPA阳性。另一类患者则会出现类似于脊柱关节炎（Spondyloarthritis, SpA）的表现，例如炎性腰背痛、附着点炎、趾炎以大关节受累为主的寡关节炎等，少数患者还有可能出现反应性关节炎、银屑病关节炎等表现；但不同的是这类患者并没有出现HLA-B27阳性^[16]^。

## 3 炎性肌病（inflammatory myopathy, IM）

与炎性关节炎相比，ICIs引起的IM少见。这类情况通常发生于使用ICIs的前两个月，常急性起病。患者临床常表现为肌痛、肌无力，并可有肌酶的升高，部分严重患者可出现横纹肌溶解、呼吸肌受累或心肌受累，并有可能危及生命。病理表现主要为局灶坏死性的肌病，并有大量的巨噬细胞和T细胞的浸润，没有皮肌炎特征性的束周肌萎缩表现。与传统IM不尽相同的是，ICIs所致IM患者罕见经典的皮肌炎相关皮疹，血清学检查提示肌炎特异性抗体通常为阴性，并有可能出现中轴部位或者面部肌肉受累^[17-22]^。少数ICIs所致IM患者还有可能合并出现炎性筋膜炎或者重症肌无力^[23, 24]^。在笔者的临床工作中，曾碰见数例患者在使用ICIs之后出现肌酸激酶明显升高，但临床并无明显肌痛、肌无力的症状。提示对于使用ICIs的患者，在临床随访过程中即使无肌痛、肌无力等症状，也应注意监测肌酶水平变化。

## 4 其他结缔组织病

肿瘤患者应用ICIs之后新发其他结缔组织病的报道相对少见。在约翰霍普金斯医院2012年-2016年接受ipilimumab和/或nivolumab治疗的实体瘤患者中，有4例患者出现了干燥综合征相关的表现，这几例患者起病相对都较急，有1例患者合并了腮腺肿大，2例患者合并出现其他irAEs，和传统干燥综合征不同的是，这几例患者的抗SSA抗体均为阴性^[14]^。此外，还有研究^[25]^报道了1例黑色素瘤患者在使用帕博利珠单抗治疗后出现典型的干燥综合征并合并亚急性共济失调感觉神经病，患者对于糖皮质激素治疗的反应不佳，在使用静脉人免疫球蛋白和利妥昔单抗后很快得到了临床方面的改善。

Mayo Clinics曾报道了两例转移性黑色素瘤患者在接受帕博利珠单抗治疗后新发系统性硬化症，其中1例为弥漫型，1例局限型^[26]^。此外偶有一些系统性血管炎例如GCA，单一器官血管炎以及肉芽肿性多血管炎（granulomatous polyvasculitis, GPA）的报道^[27-29]^。

## 5 基础风湿病的复发或加重

鉴于在很多ICIs的临床研究中常不纳入既往有风湿病的患者，因此对于既往已有风湿病的患者，在使用ICIs之后出现疾病复发或加重的相关数据并不多。在笔者的临床工作中，曾处理过1例基础有结缔组织病合并间质性肺病的胃癌患者。该患者在使用纳武利尤单抗3个疗程后出现高热、间质性肺病加重，予中等剂量激素治疗效果不明显，之后予大剂量激素联合他克莫司治疗后病情好转。在一项来自麻省总医院2011年-2018年的回顾性研究^[15]^中，共发现有6例风湿病患者在使用ICIs之后出现基础疾病的复发，其中1例RA，2例PMR，2例RA合并PMR，另有1例为银屑病关节炎。从使用ICIs到出现病情复发的中位时间为4.6周，有1例患者症状明显加重，需要加用妥珠单抗治疗后病情方可得到控制。而在另一项澳大利亚的研究^[20]^中，有12例患者既往有风湿病，其中有10例患者在使用ICIs之后出现了疾病的复发，包括4例IA和6例PMR患者。不过，风湿病患者在使用ICIs之后也有可能出现其他新发的irAEs，在一项来自梅奥的研究^[30]^中，共纳入了700例接受ICIs治疗的患者，其中16例既往有风湿病史。这16例患者在使用ICIs之后有6例出现了irAEs，但仅有1例GCA患者表现为病情复发，其他5例患者均出现了其他新发的irAEs，例如结肠炎、垂体炎等。因此，对于既往有风湿性疾病的患者，在使用ICIs之后，需密切监测基础风湿病的活动情况，以警惕病情复发或加重的可能。此外，在患者出现免疫相关的副反应之后，需进行鉴别诊断，以确定是风湿病的复发，还是出现了新发的irAEs。

## 6 免疫检查点抑制剂相关风湿方面副反应的处理

对于风湿科医生来说，在处理ICIs风湿方面副反应时，首先应与肿瘤科医师团队合作，以决定ICIs的治疗是否可继续进行。考虑到目前研究提示对于出现风湿方面ICIs的患者肿瘤方面的治疗预后相对要好^[8]^，因此从整体原则来看，若肿瘤方面治疗有效，应尽量继续ICIs治疗。若出现严重副反应，必要时需暂停或永久停用ICIs^[10]^。然而，关于激素及其他免疫抑制治疗是否会影响ICIs治疗肿瘤的效果，还需要更多大规模、前瞻性的研究来进一步评估。

对于IA的患者，若症状轻微，可先考虑应用NSAIDs治疗，若NSAIDs药物无效，则可考虑予小剂量糖皮质激素（相当于泼尼松每天10 mg-20 mg口服）；若出现中等程度的关节炎，则可考虑应用泼尼松≥20 mg每天口服，并可考虑联合应用改善病情抗风湿药物（disease-modifying anti-rheumatic drugs, DMARDs）治疗；对于严重患者，必要时激素剂量可进一步加大，若患者对激素依赖可考虑应用生物制剂例如肿瘤坏死因子（tumour necrosis factor, TNF）抑制剂的治疗^[31, 32]^。从风湿科医生传统的角度来看，若患者既往有肿瘤病史，则为使用TNF抑制剂的相对禁忌。但考虑到肿瘤患者出现严重irAEs的危险性和治疗的急迫性，在出现严重炎性关节炎时必要时仍可考虑尝试TNF抑制剂治疗。最近有一项研究^[33]^发现，在小鼠肿瘤模型中，在开始抗-CTLA-4和PD-1联合治疗前，预防性使用TNF-抑制物有助于预防irAE的发生，同时可一定程度上提高肿瘤的治疗反应，因此对于TNF抑制剂在肿瘤免疫治疗中所扮演的角色值得期待。除此之外，也有文献^[34]^报道了有患者在应用ICIs治疗后出现严重多关节炎，给予IL-6受体抗体妥珠单抗治疗后情况得到了好转。至于其他生物制剂例如IL-17、IL-12/23拮抗剂是否可能有效尚有待进一步研究。

对于ICIs相关的肌肉问题，若患者仅表现为肌痛，则可继续免疫检查点抑制剂治疗，并密切监测肌酶水平，疼痛方面可予对症治疗。对于出现肌酶轻至中度升高，但临床并无明显肌痛、肌无力症状的患者，通常在停用免疫治疗后患者肌酶水平会逐渐下降。而对于出现中至重度肌炎的患者，则需进一步完善肌电图、肌肉核磁等检查，必要时行肌肉活检，同时建议筛查肌炎相关抗体，以排除常规的炎性肌病。治疗方面，建议加用糖皮质激素治疗，根据患者肌肉受累严重程度，糖皮质激素的初始剂量可考虑为泼尼松0.5 mg/kg/d-1.0 mg/kg/d；而对于出现心肌受累或其他可能危及生命的严重状况患者，必要时可考虑应用糖皮质激素冲击治疗，并可尝试联合应用人免疫球蛋白治疗。对于激素依赖的患者，可考虑联合应用免疫抑制剂治疗^[18, 19, 22, 32]^。近期新英格兰医学杂志刚刚发表了一篇关于阿巴西普（CTLA-4激动剂）成功治疗重症ICIs诱导的心肌炎的报道，也为重症肌炎患者的治疗选择提供了新的可能^[35]^。不过，阿巴西普在这类患者中治疗的有效性和风险问题还需更多的研究证实。

最后，对于既往已有风湿病的患者，在使用ICIs之后疾病复发或加重者，首先应建议患者暂停使用免疫检查点抑制剂。风湿病方面的治疗可参考传统风湿病的治疗原则，根据患者的脏器受累严重程度决定激素及免疫抑制剂用量。

总之，免疫检查点抑制剂为肿瘤治疗打开了新的局面，随之而来的irAEs也带来了很多挑战，同时也为包括风湿科医生在内的临床医生们提供了新的研究领域。希望在将来的临床和科研工作中，我们能无论在基础还是临床方面能够有更多的进步和突破，以能够更好地为患者服务。

## References

[b1] 1Cappelli LC, Gutierrez A K, Bingham CO 3^rd^, *et al*. Rheumatic and musculoskeletal immune-related adverse events due to immune checkpoint inhibitors: a systematic review of the literature. Arthritis Care Res (Hoboken), 2017, 69(11): 1751-1763. doi: 10.1002/acr.23177

[2] Hoos A (2016). Development of immuno-oncology drugs - from CTLA4 to PD1 to the next generations. Nat Rev Drug Discov.

[3] Postow MA, Callahan MK, Wolchok JD (2015). Immune checkpoint blockade in cancer therapy. J Clin Oncol.

[4] Postow MA, Sidlow R, Hellmann MD (2018). Immune-related adverse events associated with immune checkpoint blockade. N Engl J Med.

[5] Guo Y, Walsh AM, Canavan M (2018). Immune checkpoint inhibitor PD-1 pathway is down-regulated in synovium at various stages of rheumatoid arthritis disease progression. PLoS One.

[6] Blair HA, Deeks ED (2017). Abatacept: a review in rheumatoid arthritis. Drugs.

[7] Nishimura H, Nose M, Hiai H (1999). Development of lupus-like autoimmune diseases by disruption of the PD-1 gene encoding an ITIM motif-carrying immunoreceptor. Immunity.

[8] Kostine M, Rouxel L, Barnetche T (2018). Rheumatic disorders associated with immune checkpoint inhibitors in patients with cancer-clinical aspects and relationship with tumour response: a single-centre prospective cohort study. Ann Rheum Dis.

[9] Chinese Rheumatology Association (2011). The guideline for the diagnosis and treatment of polymyalgia rheumatica and giant cell arteritis. Zhonghua Feng Shi Bing Za Zhi.

[10] Calabrese LH, Calabrese C, Cappelli LC (2018). Rheumatic immune-related adverse events from cancer immunotherapy. Nat Rev Rheumatol.

[11] Goldstein BL, Gedmintas L, Todd DJ (2014). Drug-associated polymyalgia rheumatica/giant cell arteritis occurring in two patients after treatment with ipilimumab, an antagonist of CTLA-4. Arthritis Rheumatol.

[12] Micaily I, Chernoff M (2017). An unknown reaction to pembrolizumab: giant cell arteritis. Ann Oncol.

[13] Lidar M, Giat E, Garelick D (2018). Rheumatic manifesta­tions among cancer patients treated with immune check­point inhibitors. Autoimmun Rev.

[14] Cappelli LC, Gutierrez AK, Baer AN (2017). Inflammatory arthritis and sicca syndrome induced by nivolumab and ipilimumab. Ann Rheum Dis.

[15] Mooradian MJ, Nasrallah M, Gainor JF (2019). Musculo­skeletal rheumatic complications of immune checkpoint inhibitor therapy: a single center experience. Semin Arthritis Rheum.

[16] Lee KA, Kim HR, Yoon SY (2019). Rheumatic complications in cancer patients treated with immune checkpoint inhibitors. Korean J Intern Med.

[17] Liewluck T, Kao JC, Mauermann ML (2018). PD-1 inhibitor-associated myopathies: emerging immune-mediated myopathies. J Immunother.

[18] Shah M, Tayar JH, Abdel-Wahab N (2019). Myositis as an adverse event of immune checkpoint blockade for cancer therapy. Semin Arthritis Rheum.

[19] Kadota H, Gono T, Shirai Y (2019). Immune checkpoint inhibitor-induced myositis: a case report and literature review. Curr Rheumatol Rep.

[20] Mitchell EL, Lau PKH, Khoo C (2018). Rheumatic immune-related adverse events secondary to anti-programmed death-1 antibodies and preliminary analysis on the impact of corticosteroids on anti-tumour response: A case series. Eur J Cancer.

[21] Tauber M, Cohen R, Laly P (2019). Severe necrotizing myositis associated with long term anti-neoplastic efficacy following nivolumab plus ipilimumab combination therapy. Clin Rheumatol.

[22] Ganatra S, Neilan TG (2018). Immune checkpoint inhibitor-associated myocarditis. The Oncologist.

[23] Narvaez J, Juarez-Lopez P, Lluch J (2018). Rheumatic immune-related adverse events in patients on anti-PD-1 inhibitors: fasciitis with myositis syndrome as a new complication of immunotherapy. Autoimmun Rev.

[24] Makarious D, Horwood K, Coward JI (2017). Myasthenia gravis: An emerging toxicity of immune checkpoint inhibitors. Eur J Cancer.

[25] Ghosn J, Vicino A, Michielin O (2018). A severe case of neuro-Sjögren's syndrome induced by pembrolizumab. J Immunother Cancer.

[26] Barbosa NS, Wetter DA, Wieland CN (2017). Scleroderma induced by pembrolizumab: a case series. Mayo Clin Proc.

[27] Läubli H, Hench J, Stanczak M (2017). Cerebral vasculitis mimicking intracranial metastatic progression of lung cancer during PD-1 blockade. J Immunother Cancer.

[28] Manusow JS, Khoja L, Pesin N (2014). Retinal vasculitis and ocular vitreous metastasis following complete response to PD-1 inhibition in a patient with metastatic cutaneous melanoma. J Immunother Cancer.

[29] Rutgers A, van den Brom RR (2018). Systemic vasculitis developed after immune checkpoint inhibition. Arthritis Care Res (Hoboken).

[30] Richter MD, Pinkston O, Kottschade LA (2018). Brief report: cancer immunotherapy in patients with preexisting rheumatic disease: the Mayo Clinic experience. Arthritis Rheumatol.

[31] Trinh S, Le A, Gowani S (2019). Management of immune-related adverse events associated with immune checkpoint inhibitor therapy: a mini-review of current clinical guidelines. Asia Pac J Oncol Nurs.

[b32] 32Thompson JA, Schneider BJ, Brahmer J, *et al*. Management of immunotherapy-related toxicities, version 1. 2019. J Natl Compr Canc Netw, 2019, 17(3): 255-289. doi: 10.6004/jnccn.2019.0013

[33] Perez-Ruiz E, Minute L, Otano I (2019). Prophylactic TNF blockade uncouples efficacy and toxicity in dual CTLA-4 and PD-1 immunotherapy. Nature.

[34] Kim ST, Tayar J, Trinh VA (2017). Successful treatment of arthritis induced by checkpoint inhibitors with tocilizumab: a case series. Ann Rheum Dis.

[35] Salem JE, Allenbach Y, Vozy A (2019). Abatacept for severe immune checkpoint inhibitor-associated myocarditis. N Engl J Med.

